# Application of a combined radiomics nomogram based on CE-CT in the preoperative prediction of thymomas risk categorization

**DOI:** 10.3389/fonc.2022.944005

**Published:** 2022-08-23

**Authors:** Wentao Dong, Situ Xiong, Pinggui Lei, Xiaolian Wang, Hao Liu, Yangchun Liu, Huachun Zou, Bing Fan, Yingying Qiu

**Affiliations:** ^1^ Department of Radiology, Jiangxi Provincial People’s Hospital, The First Affiliated Hospital of Nanchang Medical College, Nanchang, China; ^2^ Department of Urology, The First Affiliated Hospital of Nanchang University, Nanchang, China; ^3^ Department of Radiology, The Affiliated Hospital of Guizhou Medical University, Guiyang, China; ^4^ R&D, Yizhun Medical AI, Beijing, China

**Keywords:** thymomas, risk categorization, contrast-enhanced computed tomography, radiomic nomogram, textural features, DeLong test, calibration curve, decision curve analysis

## Abstract

**Objective:**

This study aimed to establish a combined radiomics nomogram to preoperatively predict the risk categorization of thymomas by using contrast-enhanced computed tomography (CE-CT) images.

**Materials and Methods:**

The clinical, pathological, and CT data of 110 patients with thymoma (50 patients with low-risk thymomas and 60 patients with high-risk thymomas) collected in our Hospital from July 2017 to March 2022 were retrospectively analyzed. The study subjects were randomly divided into the training set (n = 77) and validation set (n = 33) in a 7:3 ratio. Radiomics features were extracted from the CT images, and the least absolute shrinkage and selection operator (LASSO) algorithm was performed to select 13 representative features. Five models, including logistic regression (LR), support vector machine (SVM), random forest (RF), decision tree (DT), and gradient boosting decision tree (GBDT) were constructed to predict thymoma risks based on these features. A combined radiomics nomogram was further established based on the clinical factors and radiomics scores. The performance of the models was evaluated using receiver operating characteristic (ROC) curve, DeLong tests, and decision curve analysis.

**Results:**

Maximum tumor diameter and boundary were selected to build the clinical factors model. Thirteen features were acquired by LASSO algorithm screening as the optimal features for machine learning model construction. The LR model exhibited the highest AUC value (0.819) among the five machine learning models in the validation set. Furthermore, the radiomics nomogram combining the selected clinical variables and radiomics signature predicted the categorization of thymomas at different risks more effectively (the training set, AUC = 0.923; the validation set, AUC = 0.870). Finally, the calibration curve and DCA were utilized to confirm the clinical value of this combined radiomics nomogram.

**Conclusion:**

We demonstrated the clinical diagnostic value of machine learning models based on CT semantic features and the selected clinical variables, providing a non-invasive, appropriate, and accurate method for preoperative prediction of thymomas risk categorization.

## Introduction

The most common anterior mediastinal tumor is thymomas, accounting for 47% of anterior mediastinal lesions (1). The World Health Organization (WHO) classified thymic tumors in 1999. Thymomas is divided into five types: A, AB, B1, B2, and B3 according to the morphology of epithelial cells and the ratio of lymphocytes to epithelial cells (2, 3). Based on previous research, types B2 and B3 are more aggressive than types A, AB, and B1, and types B2 and B3 have lower survival rates than types A, AB, and B1 (4). Compared with types B2 and B3, types A, AB, and B1 have more chance of complete resection by operation. Thymomas of type B2 or B3 generally need neoadjuvant chemoradiotherapy (5–7). Hence, many studies have classified thymomas into low-risk (types A, AB, and B1) and high-risk groups (types B2 and B3) (8, 9), preoperative differentiation between low-risk and high-risk thymomas is significant for selecting the treatment options.

Chest CT is the preferred examination method for thymic lesions. Han. et al. (10) examined 159 patients with thymomas who underwent CT prior to operation and observed that the volume of high-risk thymomas (B2 and B3) was larger than that of low-risk thymomas (A, AB, and B1), accompanied by calcification, irregular contour, and infiltration of vascular and mediastinal fat. However, the relevant statistical difference was not significant, and the corresponding area under the curve (AUC) value was not significantly promising. CT perfusion is widely applicable in the field of oncology, which is related to the characteristics, prognosis, and therapeutic response of tumors. Yu C. et al. (11) performed an energy spectrum CT and perfusion scan on 51 patients with thymomas of different WHO subtypes and observed that the spectral parameters and perfusion of types A and AB were higher than those of other subtypes. However, the sample size of this experiment was excessively small, and the reliability of the experimental results was questioned. In recent years, positron emission tomography (PET)/CT has become increasingly important in the diagnosis of thymic malignant tumors. MFK Benveniste et al. (12) demonstrated that B3 thymoma had higher fluorodeoxyglucose uptake than other subtypes, however, PET/CT costs were higher. Owing to its efficient soft-tissue contrast resolution, magnetic resonance imaging (MRI) is more advantageous than CT in differentiating solid and cystic thymic lesions and evaluating tumor envelope and vascular, pleural, and pericardium infiltration (13, 14). However, it is still difficult to assess the type of thymoma risk. Invasive procedures, such as endoscopic biopsy, are risky owing to the proximity of anterior mediastinal tumors to the great mediastinal vessels and heart (15, 16). Therefore, preoperative acquisition of a method that can predict different risk categories of thymomas will have clinical application value.

Radiomics is a new branch of radiology that has recently emerged as an alternative to traditional qualitative diagnostic methods (17, 18). The purpose of radiomics is to identify subtle differences in radiographs that are imperceptible to the human eye. Several previous studies have suggested this novel technique for predicting thymoma risk types to overcome the limitations of CT and MRI qualitative interpretation. This study investigated the feasibility and application value of combined radiomics nomogram based on contrast-enhanced CT (CE-CT) in predicting the risk categorization of thymomas.

## Materials and methods

### Setting and participants

It was a retrospective study in which we reviewed relevant clinical features and radiological data collected from 2017 to 2022. The data were subject to rigorous review and formal acceptance by the hospital’s ethics committee. We formally obtained informed consent from all concerned individuals, especially the patient. All relevant norms and regulations agreed upon worldwide were applied in this study. Patients enrolled in this study met the following criteria: (1) No previous or current history of other malignancies except thymomas; (2) No relevant treatment was performed before preoperative mediastinal CE-CT scan; (3) The quality of the image was good without respiratory artifacts. Finally, 110 patients were considered in our study.

### Examination methods

CT images were obtained by the SOMATOM Definition CT scanner. We used these scan parameters for automatic modulation: Tube voltage, 120 kV; tube current, 150 mAs; slice thickness, 5 mm; reconstruction interval, 1 mm; slice gap, 1 mm. The CE-CT images of the arterial phase and venous phase were collected 20 and 40 seconds following the injection, respectively. The obtained CT images were uploaded to the image archiving and communication system and exported in DICOM format.

### ROI segmentation

The CE-CT venous phase images were manually segmented. Areas of interest were delineated through the DARWIN scientific research platform https://arxiv.org/abs/2009.00908 (Beijing Yizhun Intelligent Technology Co., LTD., China, https://www.yizhun-ai.com). The 3D-ROI was manually segmented by a 5-year veteran radiologist who was blinded to the risk categorization of the tumor, and the ROI was traced along the edges of the lesion, including calcification. Finally, all profiles were reviewed by another senior radiologist with >15 years of experience. If an ROI segmented by two radiologists were inconsistent, i.e., Intersection over Union (IoU) <=95%, the lesion boundary was determined by the senior radiologist.

### Feature extraction and selection in radiomics

**Figure 1:**
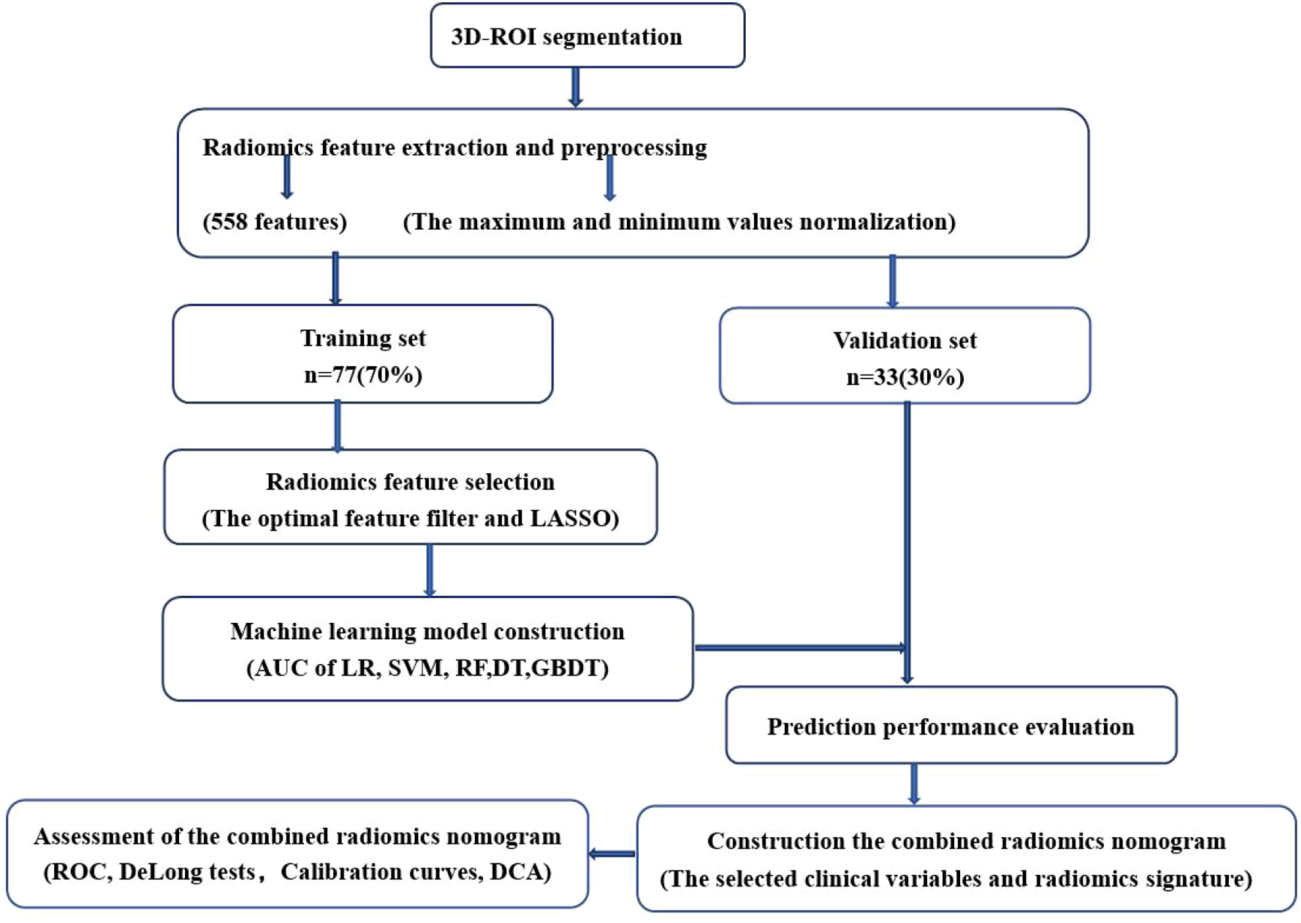
Radiomics workflow.

**Figure 2:**
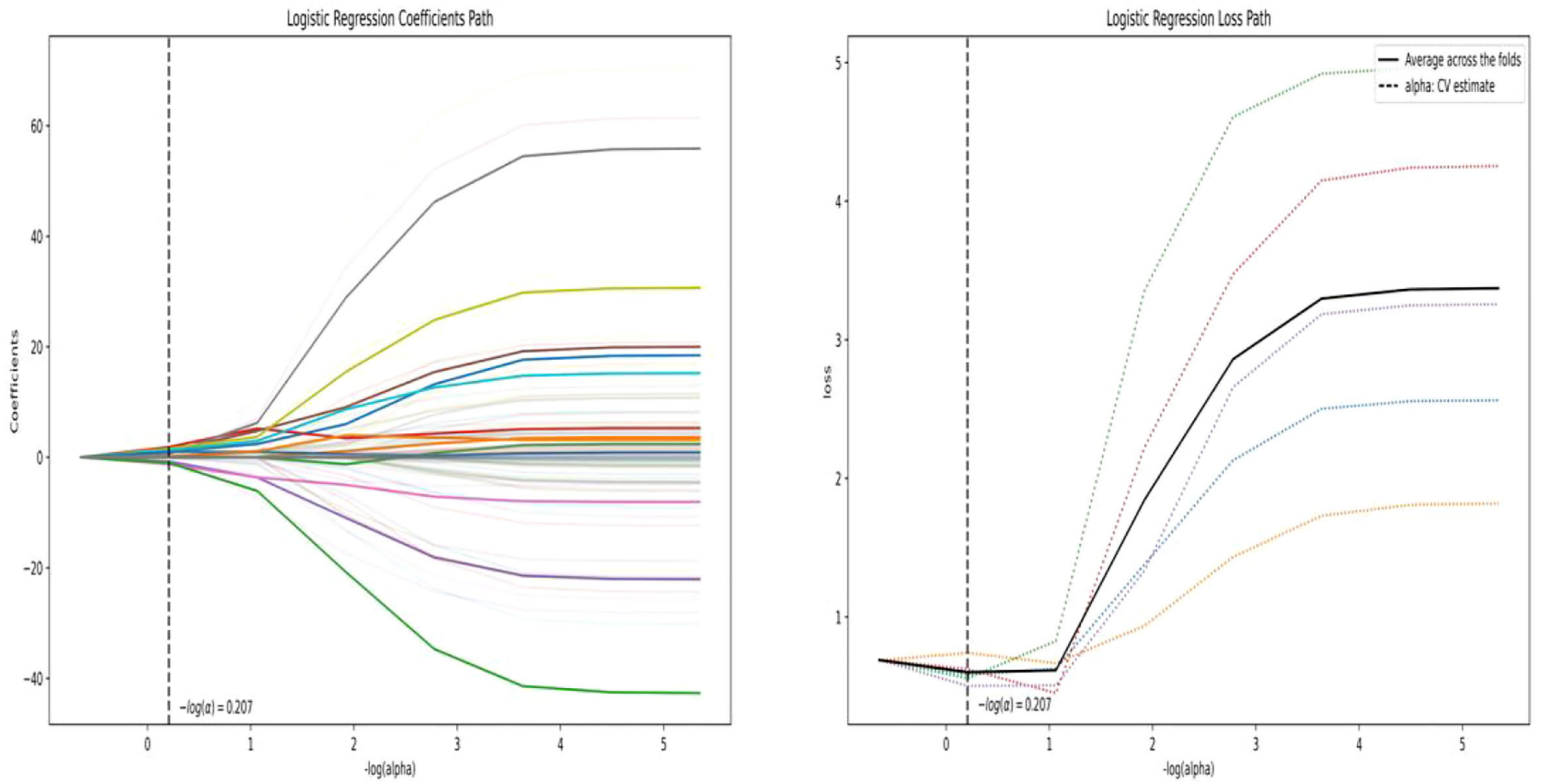
ASSO algorithm for feature selection. LASSO path (left); MSE path (right).

**Figure 3:**
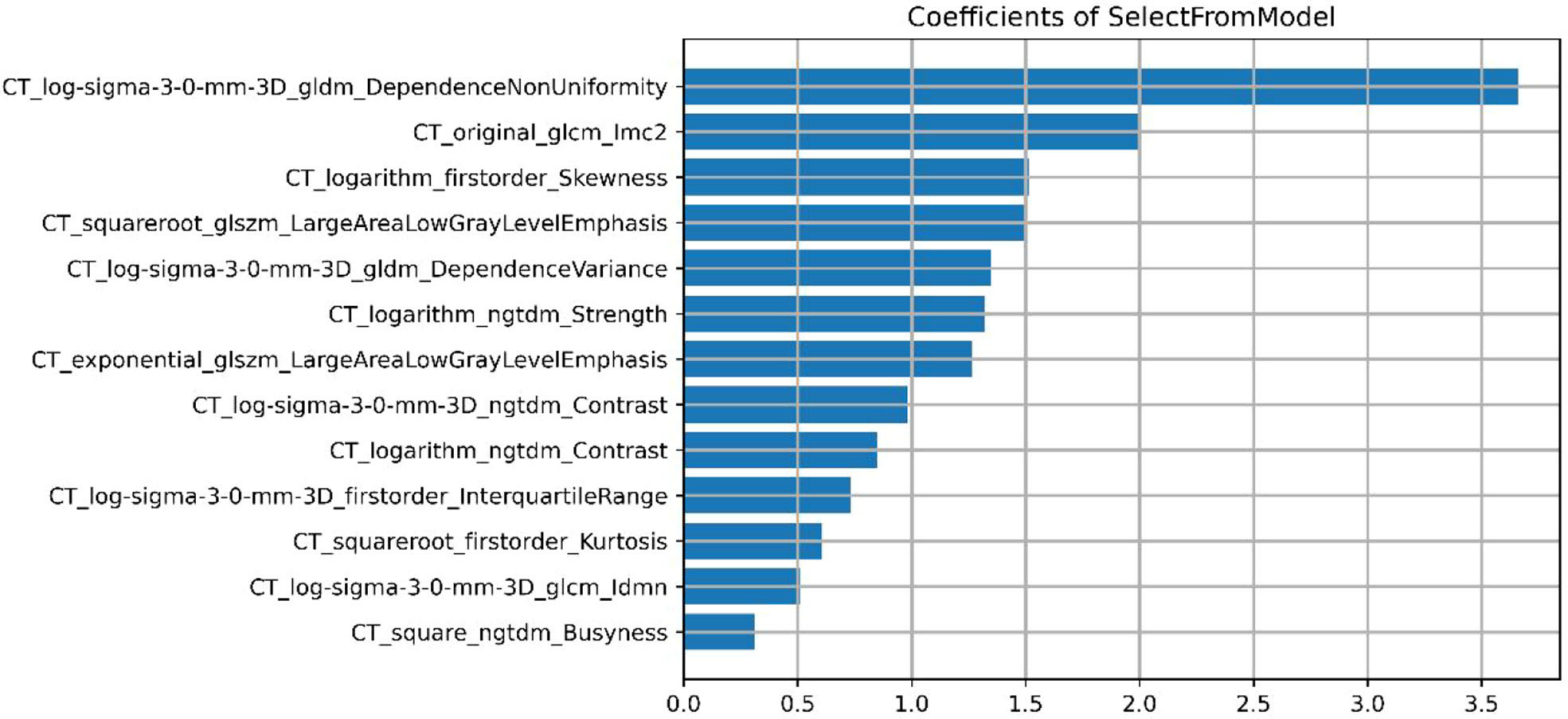
The final selected 13 features (3 first-order statistical features and 10 textures).

A total of 558 radiomics features were extracted using the above-mentioned platform, including first-order statistical features, texture features, and shape-based 3D features (**Figure 1**). For the classification, normalized minimum and maximum values were used to linearly stretch the properties of each dimension to an interval (1). To make the algorithm converge faster and receive a more reasonable model, we preprocessed the data. The computer-generated data set was randomly assigned, of which 70% of the data set was assigned to the training set (35 low-risk groups and 42 high-risk groups) and 30% to the validation set (15 low-risk groups and 18 high-risk groups). We used feature selection in classifier training, which plays a very important role. The linear correlation between the category label and each feature was assessed by an optimal feature filter (i.e., sample variance F value) (19), and the 101 most relevant features were selected from 558 features. To further select the optimal prediction feature from the above-mentioned features, we used the LASSO algorithm (**Figure 2**), and 13 of the most relevant features for thymomas typing were obtained, including three first-order statistical features and ten textures (**Figure 3**).

### Radiomics signature construction

The optimal subset was selected by reducing the proportion. Five machine learning models, including logistic regression (LR), support vector machine (SVM), random forest (RF), decision tree (DT), and gradient boosting decision tree (GBDT) were constructed. Using the same threshold set in the training set, the prediction performance of the five models in the independent validation set was further tested. The accuracy of the model was verified by the 10-fold cross-validation method. Receiver operating characteristics (ROC) and its corresponding AUC were applied to evaluate the performance of the above-mentioned models, and the sensitivity, specificity, and accuracy of these models were also calculated. The optimal efficient model was selected from the five machine learning models.

### Development and assessment of combined radiomics nomogram

To screen for independent predictors of thymoma in the low-risk and high-risk groups, we used multivariate logistic regression analysis that included potential predictors such as clinical risk factors and imaging features. To distinguish low-risk thymomas from high-risk thymomas, a combined radiomics nomogram was constructed based on the selected variables. A quantified combined radiomics nomogram was identified using a formula derived from the training set to calculate the radiomics score for each patient in the validation set. In addition to the AUC calculations, DeLong tests and calibration curves were performed. Finally, the clinical value of this combined radiomics nomogram was evaluated by DCA.

### Statistical analysis

R statistical tool (Version 3.4.4) and SPSS 19.0 software were used for analysis. Statistical data were compared among the two groups by the χ^2^ test. T-test (normal distribution) and Mann−Whitney U test (skewness distribution) were used to statistically compare the probability scores between low-risk and high-risk thymomas. AUC value, accuracy, sensitivity, and specificity were calculated to evaluate the predictive performance of the model (20).

## Results

### Patient characteristics

There were no significant differences in age, sex, myasthenia gravis, maximum tumor diameter, calcification, boundary, and pleural effusion factors between patients in the training set and the validation set, as indicated in **Table 1** (P*-value > 0.05 for all). Nevertheless, for patients with low-risk and high-risk thymomas, significant statistical differences were observed in maximum tumor diameter and boundary (P-value < 0.05), as presented the results of the multivariate logistic regression analysis in **Table 2**, other differences were not significant.

**Table 1:** Demographic characteristics in the training and validation set.

	Training set (n=77)		P-value	Validation set (n = 33)	P*-value
	Low-risk	high-risk			
**Age**	37.97 ± 13.15	49.43 ± 14.24	<0.001	43.21 ± 11.95	0.730
**Sex**			0.833		1.000
female	15(19.48%)	17(22.08%)		14(42.4%)	
male	20(25.97%)	25(32.47%)		19(57.6%)	
**MG**			0.864		0.899
No	21(27.27%)	26(33.77%)		19(57.6%)	
Yes	14(18.18%)	16(20.78%)		14(42.4%)	
**MTD**	34.37 ± 14.62	51.73 ± 15.72	<0.001	41.51 ± 15.02	0.506
**Calcification**			0.006		0.738
No	26(33.76%)	18(23.38%)		17(51.5%)	
Yes	9(11.69%)	24(31.17%)		16(48.5%)	
**Boundary**			<0.001		0.672
clear	24(31.17%)	9(11.69%)		12(36.4%)	
obscure	11(14.29%)	33(42.85%)		21(63.6%)	
**Pleural effusion**			<0.001		1.000
No	29(37.66%)	18(23.38%)		20(60.6%)	
Yes	6(7.79%)	24(31.17%)		13(39.1%)	
**Rad-score**	0.31 ± 0.17	0.71 ± 0.23	<0.001	0.62 ± 0.28	0.138

MG, Myasthenia gravis; MTD, Maximum tumor diameter (mm, x¯ ± s).

P-value < 0.05: significant difference between low-risk and high-risk group in the training set.

P*-value < 0.05: significant difference between training and validation set.

**Table 2:** Results of univariate and multivariate logistic regression for predicting the risk categorization of thymomas.

Variable	Univariate regression			Multivariate regression		
	Odds ratio	(95% CI)	*P*-value	Odds ratio	(95% CI)	*P*-value
Age	0.93	[0.90;0.96]	<0.001	0.98	[0.94;1.03]	0.523
Sex	0.99	[0.46;2.11]	0.972	NA	NA	NA
MG	1.16	[0.54;2.51]	0.696	NA	NA	NA
MTD	0.95	[0.92;0.97]	<0.001	0.96	[0.93;0.99]	0.016
Calcification	0.28	[0.12;0.62]	0.002	0.98	[0.34;2.83]	0.965
Boundary	0.12	[0.05;0.28]	<0.001	0.24	[0.07;0.80]	0.020
Pleural effusion	0.17	[0.07;0.41]	<0.001	0.70	[0.20;2.42]	0.573

MG, Myasthenia gravis; MTD, Maximum tumor diameter (mm, x¯ ± s).

### Prediction performance of machine learning models

**Figure 4:**
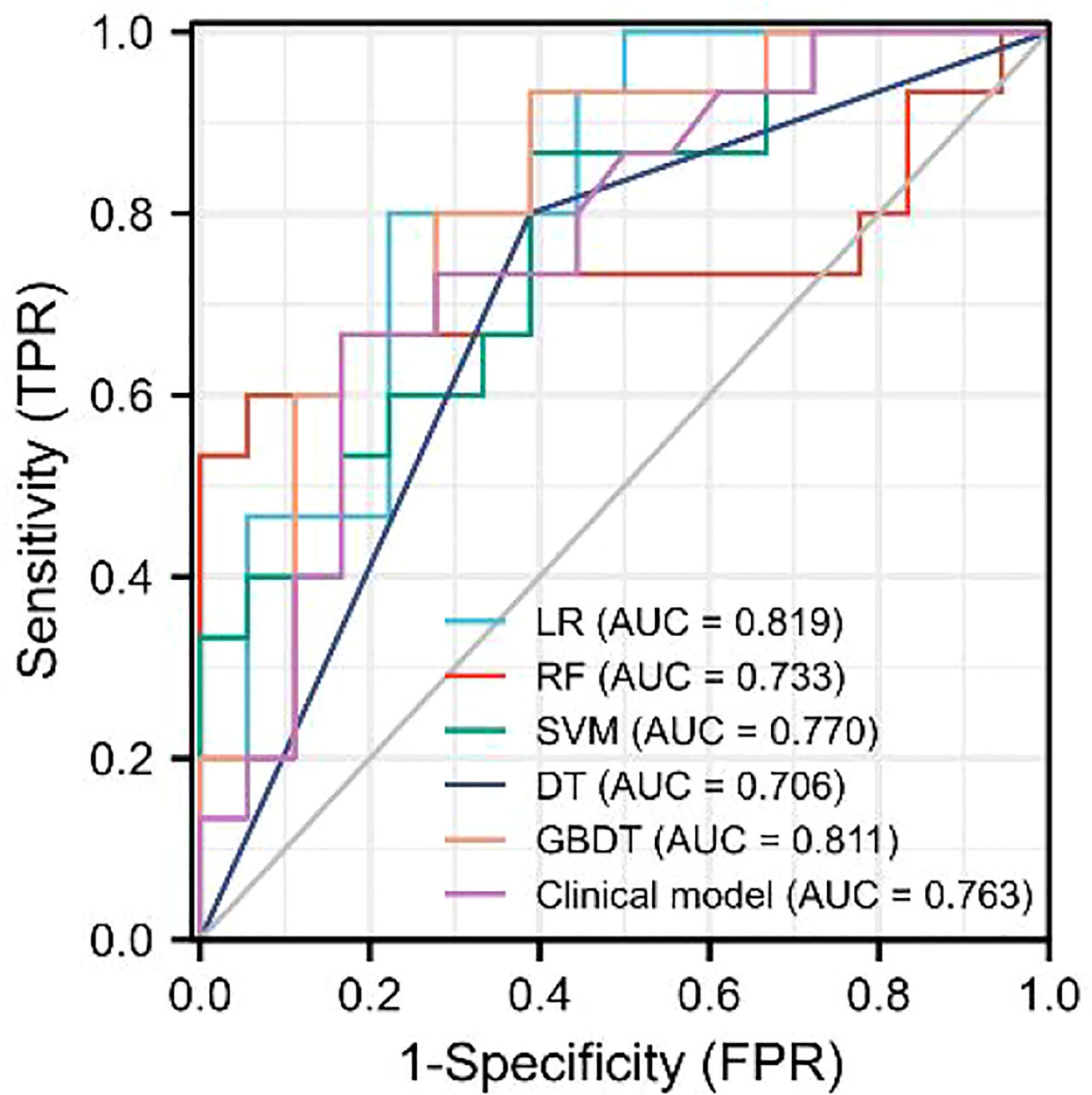
ROC curves of the five machine learning models and clinical model in the validation set.

**Figure 5:**
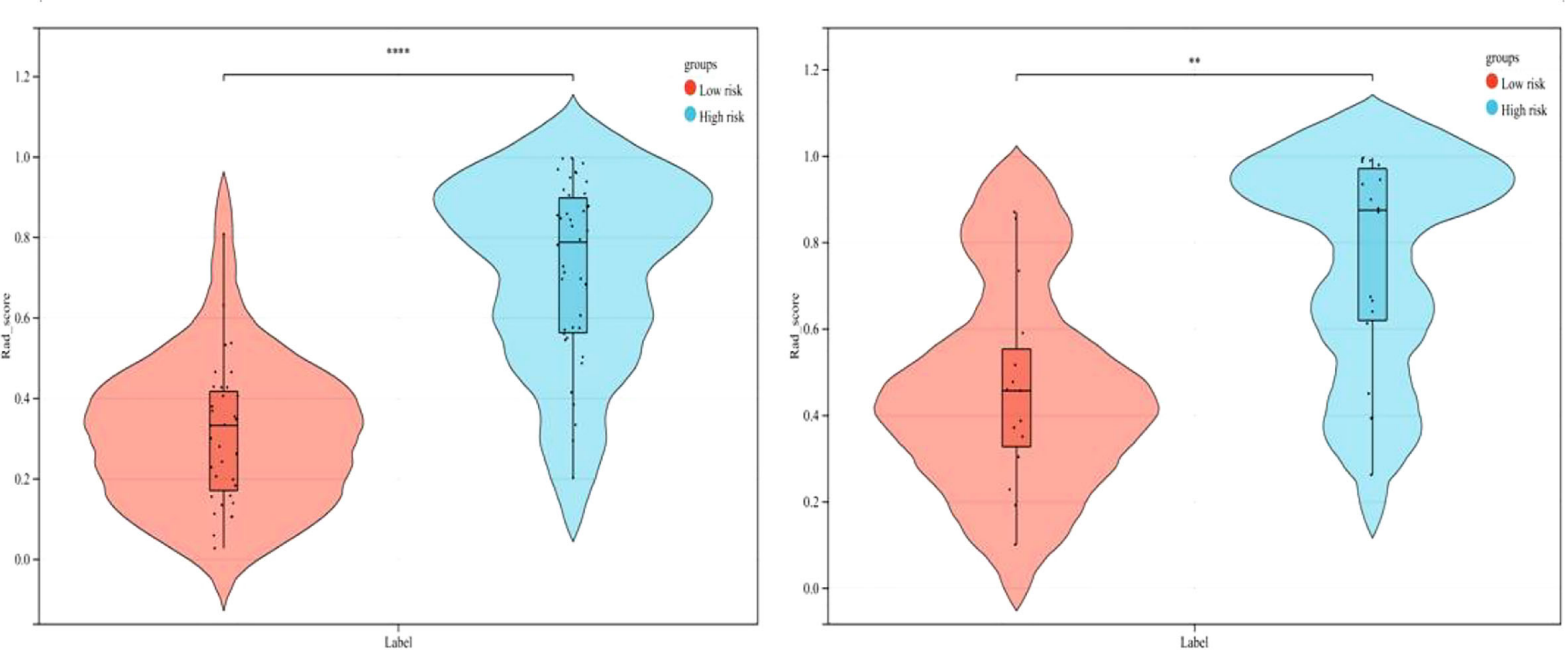
Comparison of radiomics score between low-risk and high-risk thymomas in the training and validation sets (left, training set; right, validation set).

As demonstrated in **Table 3**, the AUC of LR, SVM, RF, DT, and GBDT were 0.910, 0.897, 1.000, 1.000, and 1.000 in the training set, respectively. The AUC of LR, SVM, RF, DT, and GBDT were 0.819, 0.770, 0.733, 0.706, and 0.811 in the validation set, respectively. The ROC curves of the five machine learning models and clinical model are exhibited in **Figure 4**. DT and RF have poor prediction effects on thymomas risk categorization whereas the other machine learning models had relatively high prediction effects. The LR was the best radiomics model that performed most efficiently in the validation set. The AUC, accuracy, sensitivity, and specificity were 0.819 (95% CI: 0.670–0.960), 0.788, 0.778, and 0.800, respectively. Cross-validation was performed within the training set to get a set of best hyperparameters. During training and testing, radiomics scores of both low-risk and high-risk samples demonstrated significant statistical differences, as presented in **Figure 5**. This indicated the radiomics signature was closely related to thymomas risk categorization.

**Table 3:** Predictive performance of training and validation sets for five machine learning models.

	Training set				Validation set			
	ACC	SEN	SPE	AUC	ACC	SEN	SPE	AUC
**LR** **SVM** **RF** **DT** **GBDT**	0.857 0.818 1.000 1.000 1.000	0.809 0.809 1.000 1.000 1.000	0.914 0.829 1.000 1.000 1.000	0.910 0.897 1.000 1.000 1.000	0.788 0.697 0.788 0.697 0.727	0.778 0.611 0.944 0.611 0.611	0.800 0.800 0.600 0.800 0.867	0.819 0.770 0.733 0.706 0.811

### Combined radiomics nomogram

**Figure 6:**
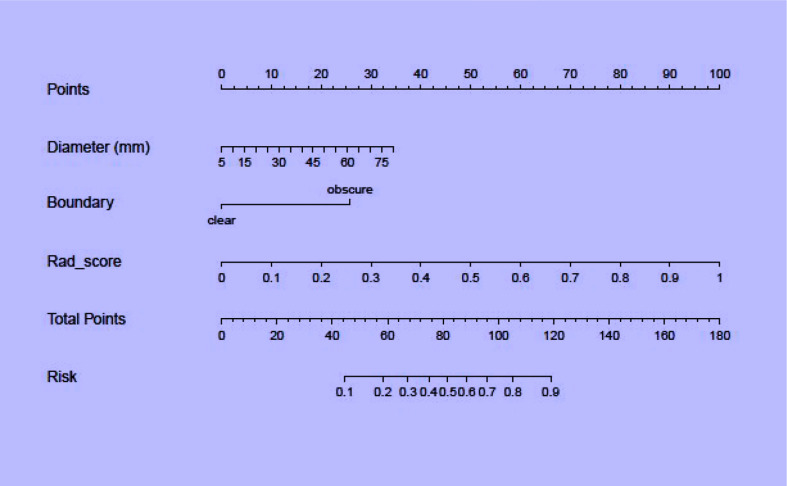
A combined radiomics nomogram for predicting thymomas risk categorization.

Regarding clinical variables, after multivariate logistic regression analysis, only maximum tumor diameter and boundary represented independent predictive variables of low-risk and high-risk thymomas. Then, the clinical model was developed based on the above independent variables and validated in the validation set. The AUC in the training and validation sets was 0.835 and 0.763, respectively. To develop a more precise and clinically applicable model to predict thymomas risk categorization, we used the LR algorithm to construct a combined radiomics nomogram incorporating CE-CT radiomic features, maximum tumor diameter and boundary as presented in **Figure 6**.

**Figure 7:**
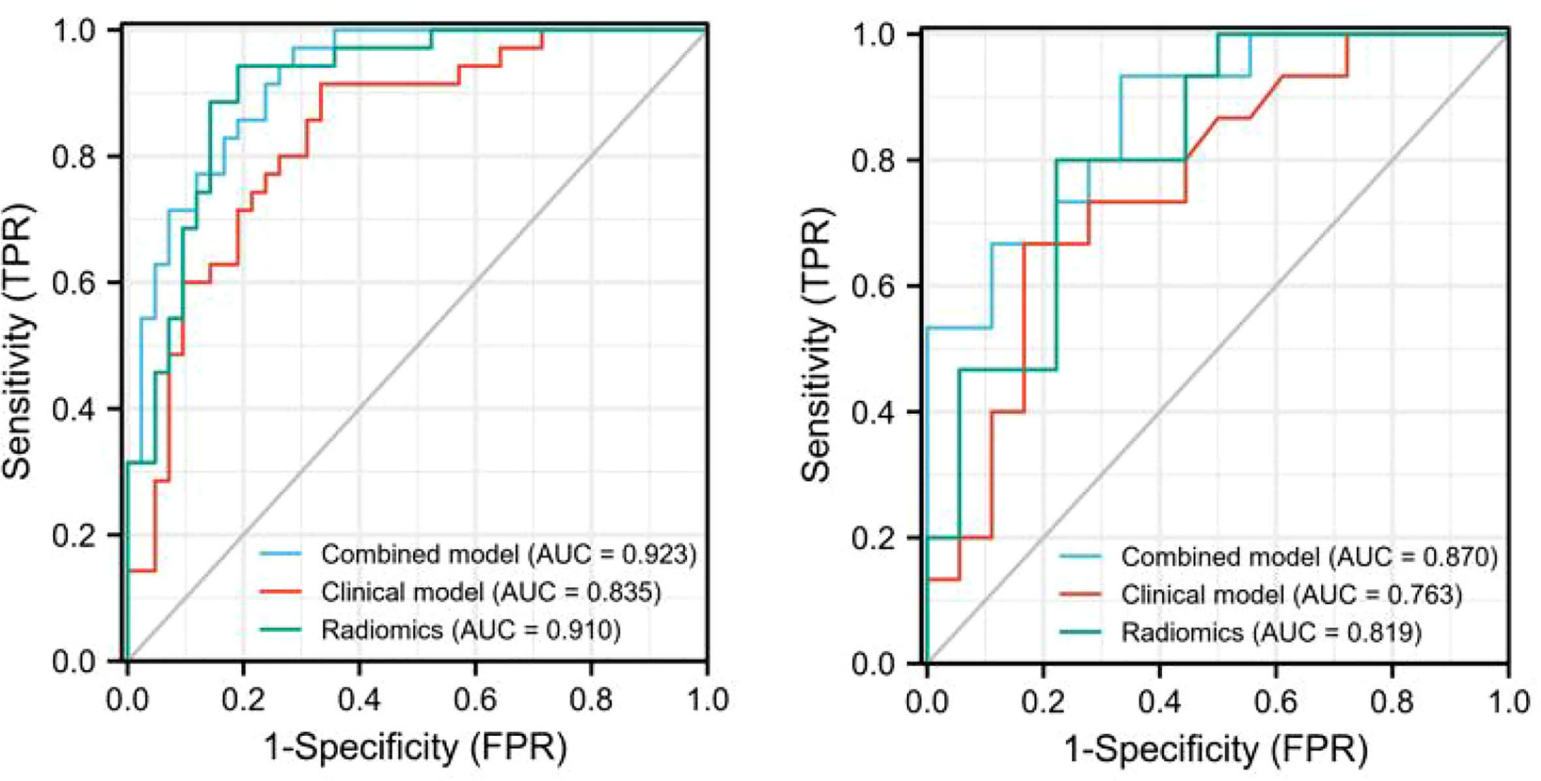
The AUC values for combined radiomics nomogram, clinical and radiomics were used to differentiate between low-risk and high-risk thymomas (Left, training set; right, validation set).

**Figure 8:**
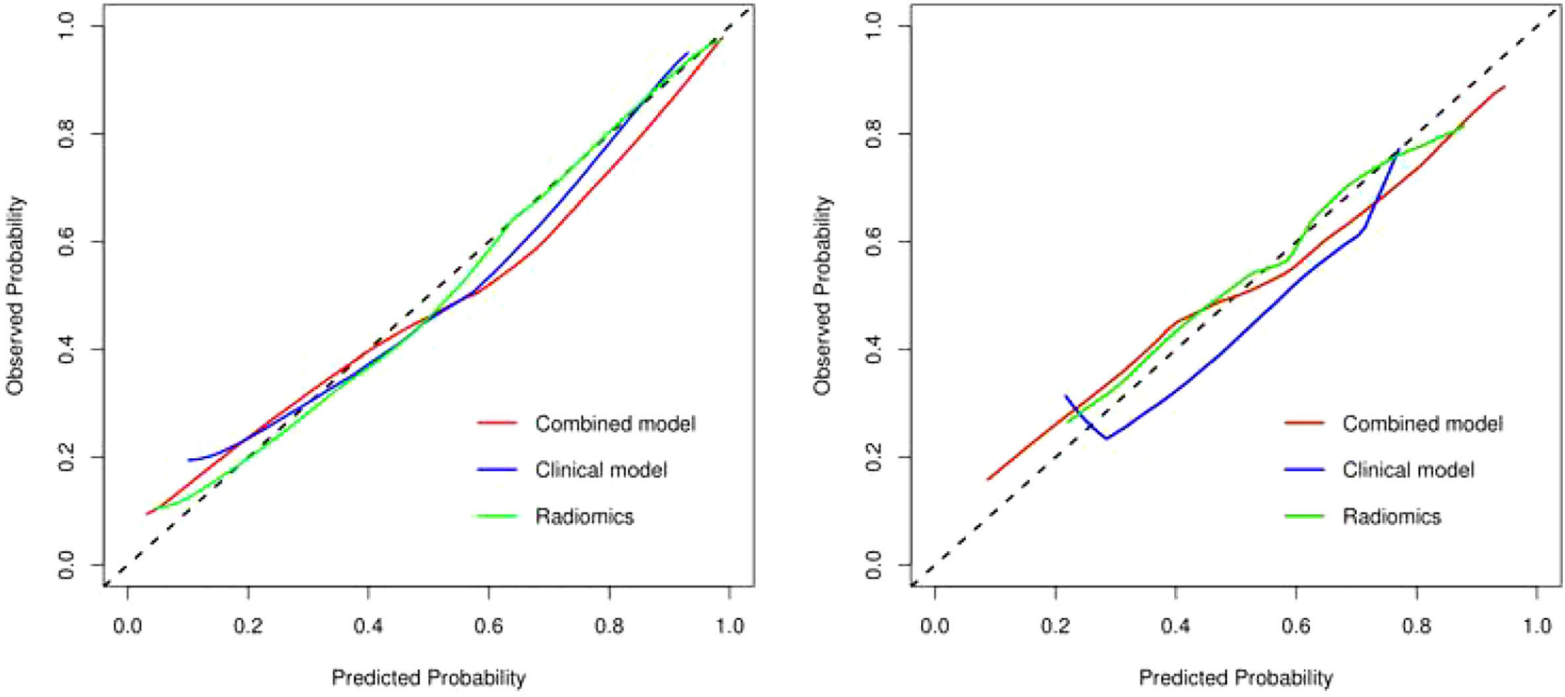
The calibration curve analysis of combined radiomics nomogram, clinical, and radiomics models (left, training set; right, validation set). The 45° line represents a perfect match between the actual (Y-axis) and the probability of differential diagnosis combined with combined radiomics nomogram, clinical, and radiomics prediction (X-axis). The closer the distance between the two curves, the higher the accuracy of prediction and actual observation of thymomas risk categorization. .

**Figure 9:**
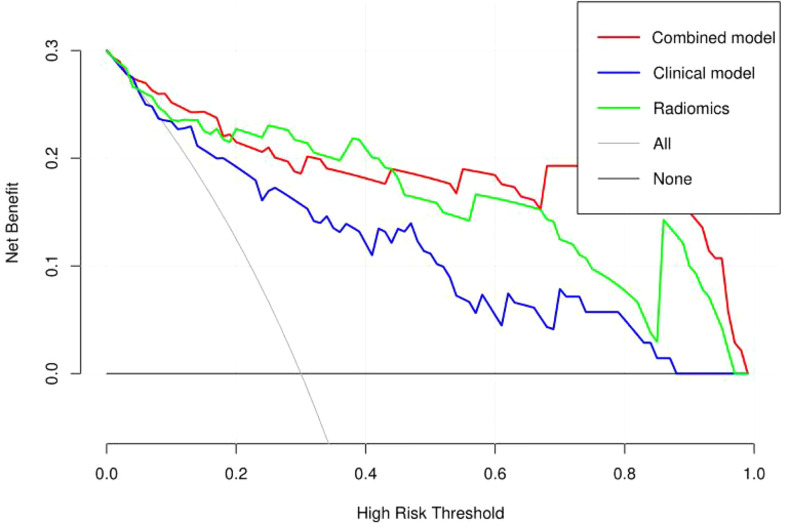
Decision curve analysis (DCA) of the combined radiomics nomogram, clinical, and radiomics models in the combined training and validation set.

The discriminating efficacy of the combined radiomics nomogram was confirmed in the ROC analysis with an AUC of 0.923 for the training set and 0.870 for the validation set, respectively (**Figure 7**). The AUC value of the training and validation sets was higher than that of clinical and radiomics model. According to the DeLong test, although p value between combined model and clinical model was not less than 0.05 in the validation set, it was already the smallest among several models, it indicated the combined radiomics nomogram has the strongest significance compared with other models in the risk type assessment of thymoma (**Table 4**). Excellent consistency among the predicted and actual thymomas risk categorization was presented using the calibration curves of the combined radiomics nomogram, clinical, and radiomics models (**Figure 8**). DCA exhibited that the combined radiomics nomogram had the greatest clinical utility, with a threshold probability of >5%, suggesting that the combined radiomics nomogram was a reliable clinical tool for predicting the thymomas risk categorization (**Figure 9**).

**Table 4:** Comparison of the prediction with the combined radiomics nomogram, clinical, and radiomics model.

Group	Model 1	Model 2	*P*-value
Training	Combined	Clinical	0.024
	Combined	Radiomics	0.482
	Clinical	Radiomics	0.157
Validation	Combined	Clinical	0.142
	Combined	Radiomics	0.365
	Clinical	Radiomics	0.632

## Discussion

To overcome the limitations of qualitative interpretation of CT and MRI studies, some recent studies have explored the application of radiomics in diagnosing thymic lesions. Gang Xiao et al. (21) reported that the combined radiomics nomogram can be used for individual diagnosis of thymomas and subtype prediction, however, only the LR machine learning model was used to develop a combined radiomics nomogram and the possibility of other learning models was not analyzed. Kayicangir A et al. (22) selected four radiomics models to distinguish low-risk thymomas from high-risk thymomas but did not further explore the value of the combined radiomics nomogram. Therefore, based on five machine learning models, this study selected the best model combined with relevant clinical features and made a combined radiomics nomogram to better predict the risk type of thymomas. We demonstrated the classification efficiency of the radiomics model can be further improved by adding clinical features. The radiomics signature could only reflect the information presented in CE-CT images, while the clinical features provided other clues identifying the risk of the disease. A combination of both factors yielding the best performance in our experiments.

The relevant clinical features selected in this study were the maximum tumor diameter and boundary, which were revealed through multivariate logistic regression analysis; however, other features did not exhibit statistically significant differences to predict the risk categorization of thymomas (**Table 2**). The reason for this is that low-risk thymomas masses are usually small, regular, or superficial lobulated, with uniform density, small cystic changes, septum and margin calcification, intact capsule, and clear fat stranding. When the mass is large, it can compress but does not invade surrounding structures, thus, the boundary is clear. In contrast, high-risk thymomas are usually large masses with lobulated shapes, irregular edges, and uneven internal density or signals. Specular calcification, cystic degeneration, and necrosis are common in tumors. The surrounding fat space may be partially narrowed or disappeared, accompanied by ipsilateral pleural implantation metastasis and invasion or progression of the pericardium and great vessels (10, 23). Pleural effusion can occur in both low-risk and high-risk thymomas, it is common in patients with invasive thymomas. However, as presented the results of the multivariate logistic regression analysis in **Table 2**, pleural effusion was not a predictive risk type for thymomas. This result is the same as Gang Xiao et al. (21). The reason may be the relatively small number of cases.

In this study, a combined radiomics nomogram based on CE-CT was established and validated to quantify the probability of a differential diagnosis of low-risk and high-risk thymomas. This is a noninvasive, fast, and convenient method. Venous CE-CT images were selected for feature extraction, and CE-CT in the venous phase reflects a larger number of new dysfunctional vessels. The density and permeability of these vessels are higher, which makes a higher number of contrast agents stay in the interstitial space of tumor cells, and the enhancement of lesions is more abundant, comprehensively highlighting the heterogeneity and biological characteristics of the tumor. Chen et al. (24) also confirmed this. Feature extraction was sketched using 3D-ROI, and 13 radiomics features were selected for identification (**Figure 3**). The best prediction radiomics model was obtained by LR, presenting an AUC value of 0.819 in the dependent validation set (**Table 3**. **Figure 4**). This was different from the results provided by Feng et al. (25). The results of their experiments revealed that the SVM model has the best predictive performance for the simplified thymoma risk categorization, and this performance was better than the radiologist’s assessment. The reason may be that they used 2D-ROI segmentation to extract features whereas we used 3D-ROI segmentation. We provided 3D texture information about the tumor, and the LR model is better at capturing this (26). The combined radiomics nomogram was constructed by incorporating the radiomics score and relevant clinical features, whose AUC value is 0.923 for the training set and 0.870 for the validation set, higher than clinical and radiomics model **(** **Figure 7** **)**. The combined radiomics nomogram has better ability than clinical risk factors and radiomics features alone in predicting the risk type of thymomas. This suggests that a combined radiomics nomogram, based on the limited sample information, can seek an optimal compromise solution between model complexity and learning ability to obtain the optimal generalization ability. It has a unique advantage in resolving small sample sizes, high dimensions, and nonlinearity (27). The nomogram prediction model has been broadly applied in clinical medicine recently (28). Risk scores are used to represent risk factors that predict prognosis for various diseases. The model is concise and easy to comprehend and operate, which is beneficial for doctor-patient communication.

Our combined radiomics nomogram based on the radiomics and clinical features of CE-CT can accurately predict thymomas of different risk categorizations. LASSO algorithm was applied to finally select 13 types of radiomics feature parameters in this study, which contained simple morphological and more comprehensive higher-order features (29). Gray Level Dependence Matrix (GLDM) had the highest Maximum Relevance Minimum Redundancy score in differentiating low-risk from high-risk thymomas. GLDM presents higher-order texture features, and its extended method can obtain the second-order or higher-order statistics of the relationship between the gray values of pixel pairs or pixel sets to estimate their probability-density function. Its effectiveness has been confirmed by many studies (30–32). However, most of the radiomics features are not well-combined with physiology and pathology in the current research. Although the machine learning model established by the above-mentioned partial radiomics features can achieve the desired research purpose well, only a few studies have clarified the role of these features in the model and the biological mechanism behind them. Its potential significance should be further studied in the future.

There are some limitations to our study. First, only one medical center participated in the study, and the number of cases was relatively small. The multi-center collaborative study is still needed, with additional cases to reduce sample selection bias and regional differences. Second, manual lesion delineation may lead to errors, thus, losing partial image information. Therefore, more accurate lesion contour delineation methods, such as semi-automatic segmentation, are needed to extract the characteristic values of the lesion.

Conclusively, the LR model based on radiomics feature extraction from CE-CT data is a non-invasive tool with well predictive accuracy and stability, which can help predict the risk classification of thymomas prior to operation. The combined radiomics nomogram model can improve the ability to predict thymomas risk categorization. This method can be used as a preoperative technique to determine the surgical approach for thymomas.

## Data availability statement

The original contributions presented in the study are included in the article/supplementary materials. Further inquiries can be directed to the corresponding authors.

## Ethics statement

The studies involving human participants were reviewed and approved by the ethics committee of the Jiangxi Provincial People’s Hospital. The patients/participants provided their written informed consent to participate in this study.

## Author contributions

The authors made the following contributions: WD and BF made the conception for this research. Data collection and analysis were performed by WD, PL, XW, YQ, HZ and SX. WD, PL and HL analyzed the data and drafted the article. BF, PL, SX and YL reviewed/edited the manuscript. All the authors critically revised the article for important intellectual content. All authors contributed to the article and approved the submitted version.

## Conflict of interest

Author HL was employed by company Yizhun Medical AI.

The remaining authors declare that the research was conducted in the absence of any commercial or financial relationships that could be construed as a potential conflict of interest.

## Publisher’s note

All claims expressed in this article are solely those of the authors and do not necessarily represent those of their affiliated organizations, or those of the publisher, the editors and the reviewers. Any product that may be evaluated in this article, or claim that may be made by its manufacturer, is not guaranteed or endorsed by the publisher.
